# Lil3 Assembles with Proteins Regulating Chlorophyll Synthesis in Barley

**DOI:** 10.1371/journal.pone.0133145

**Published:** 2015-07-14

**Authors:** Astrid Mork-Jansson, Ann Kristin Bue, Daniela Gargano, Clemens Furnes, Veronika Reisinger, Janine Arnold, Karol Kmiec, Lutz Andreas Eichacker

**Affiliations:** Center for Organelle Research, University of Stavanger, Stavanger, Norway; Arizona State University, UNITED STATES

## Abstract

The light-harvesting-like (LIL) proteins are a family of membrane proteins that share a chlorophyll *a*/*b*-binding motif with the major light-harvesting antenna proteins of oxygenic photoautotrophs. LIL proteins have been associated with the regulation of tetrapyrrol biosynthesis, and plant responses to light-stress. Here, it was found in a native PAGE approach that chlorophyllide, and chlorophyllide plus geranylgeraniolpyrophosphate trigger assembly of Lil3 in three chlorine binding fluorescent protein bands, termed F1, F2, and F3. It is shown that light and chlorophyllide trigger accumulation of protochlorophyllide-oxidoreductase, and chlorophyll synthase in band F3. Chlorophyllide and chlorophyll esterified to geranylgeraniol were identified as basis of fluorescence recorded from band F3. A direct interaction between Lil3, CHS and POR was confirmed in a split ubiquitin assay. In the presence of light or chlorophyllide, geranylgeraniolpyrophosphate was shown to trigger a loss of the F3 band and accumulation of Lil3 and geranylgeranyl reductase in F1 and F2. No direct interaction between Lil3 and geranylgeraniolreductase was identified in a split ubiquitin assay; however, accumulation of chlorophyll esterified to phytol in F1 and F2 corroborated the enzymes assembly. Chlorophyll esterified to phytol and the reaction center protein psbD of photosystem II were identified to accumulate together with psb29, and APX in the fluorescent band F2. Data show that Lil3 assembles with proteins regulating chlorophyll synthesis in etioplasts from barley (Hordeum vulgare L.).

## Introduction

Angiosperm seedlings that germinate in the dark etiolate. Proplastids develop into etioplasts and Protochlorophyllide (Pchlide), Pchlide oxidoreductase A (PORA) and thylakoid lipids accumulate [1, 2] in an inner membrane system of prolamellar bodies that develop a semicrystalline structure [3]. The first molecular indication of the plants morphological switch from skoto- to photo-morphogenesis is the light-dependent photoreduction of Pchlide to Chlide by POR (EC 1.3.1.33), and the accumulation of Chl_GG_ and Chl_PY_ after esterification by CHS (EC 2.5.1.62) [4–7]. During deetiolation, flat membrane sacs emerge that transform into the thylakoid containing the photosynthetic complexes [8].

In photosynthesis, a family of light harvesting complex (LHC) proteins regulates harvesting of light energy and photoprotection. Members of the LHC protein superfamily include LHC proteins, LHC-like proteins, subunit S of photosystem II (PsbS), ferrochelatase II and red lineage chlorophyll a/b-binding (CAB)-like proteins (RedCAP) [9, 10]. All LHC-proteins are nuclear-encoded, bind pigments, such as chlorophyll (Chl) and carotenoids, have one to four membrane-spanning helices, are involved in light absorption and are characterized by at least one LHC motif [9].

The two-helix light-harvesting-like protein 3 (Lil3) has been associated with Chl and tocopherol synthesis in thale cress (*Arabidopsis thaliana)* [11]. In a lil3:1 and lil3:2 double mutant, Chl yield was low and Chl was exclusively found esterified to geranylgeraniol (GG) (Chl_GG_). Decrease in α-tocopherol, and loss of the enzyme geranylgeranyl reductase (GGR, EC1.3.1.83) led to the conclusion that Lil3 stabilizes GGR against degradation [11]. Recently, transmembrane amino acids in the LHC-motif of Lil3 were reported to anchor GGR to the membrane, and to be responsible for oligomerization of GGR [12]. However, recombinant plant GGR did not require Lil3 for reduction of GGPP to PYPP [12]. GGR catalyzes the NADPH dependent three-step reduction of the pyrophosphate (PP) form of GG or its esterified form in Chl_GG_. End products are phytyl pyrophosphate (PYPP) or Chl_PY_ [7]. The enzyme operates at the branching point between Vitamin E and Chl synthesis. In vitamin E biosynthesis, PYPP and homogentisate are the substrates for homogentisate phytyltransferase (HPT1, AT2G18950) and hence for tocopherol biosynthesis [13–15]. In Chl biosynthesis, Chl_PY_ provides the entry point for assembly of the Chl binding protein complexes of the photosynthetic machinery.

In *Synechocystis* PCC6803, a complex of Chl-synthase (CHS) and the high-light-inducible protein HliD, a one-helix Lil homolog, together with Ycf39 and YidC was characterized [16]. HliD was not found to be essential for the function of CHS. However, deletion of HliD increased the Chlorophyllide (Chlide) content in *Synechocystis* about 6-fold, and authors concluded that HliD could have a function in directing the assembly of other proteins like Ycf39 into a complex with CHS [16]. In barley, Lil3 was identified as the first protein to bind Chl during deetiolation [17].

Here, the Chlide and GGPP dependent assembly of Lil3 was investigated in etioplasts from barley. Protein and pigment analysis show that Chlide triggers Lil3 assembly with POR and CHS and that Chlide esterification initiates GGR and photosystem assembly.

## Results

### Assembly of Lil3 correlates with the accumulation of Chlide and ChlGG

**Fig 1:**
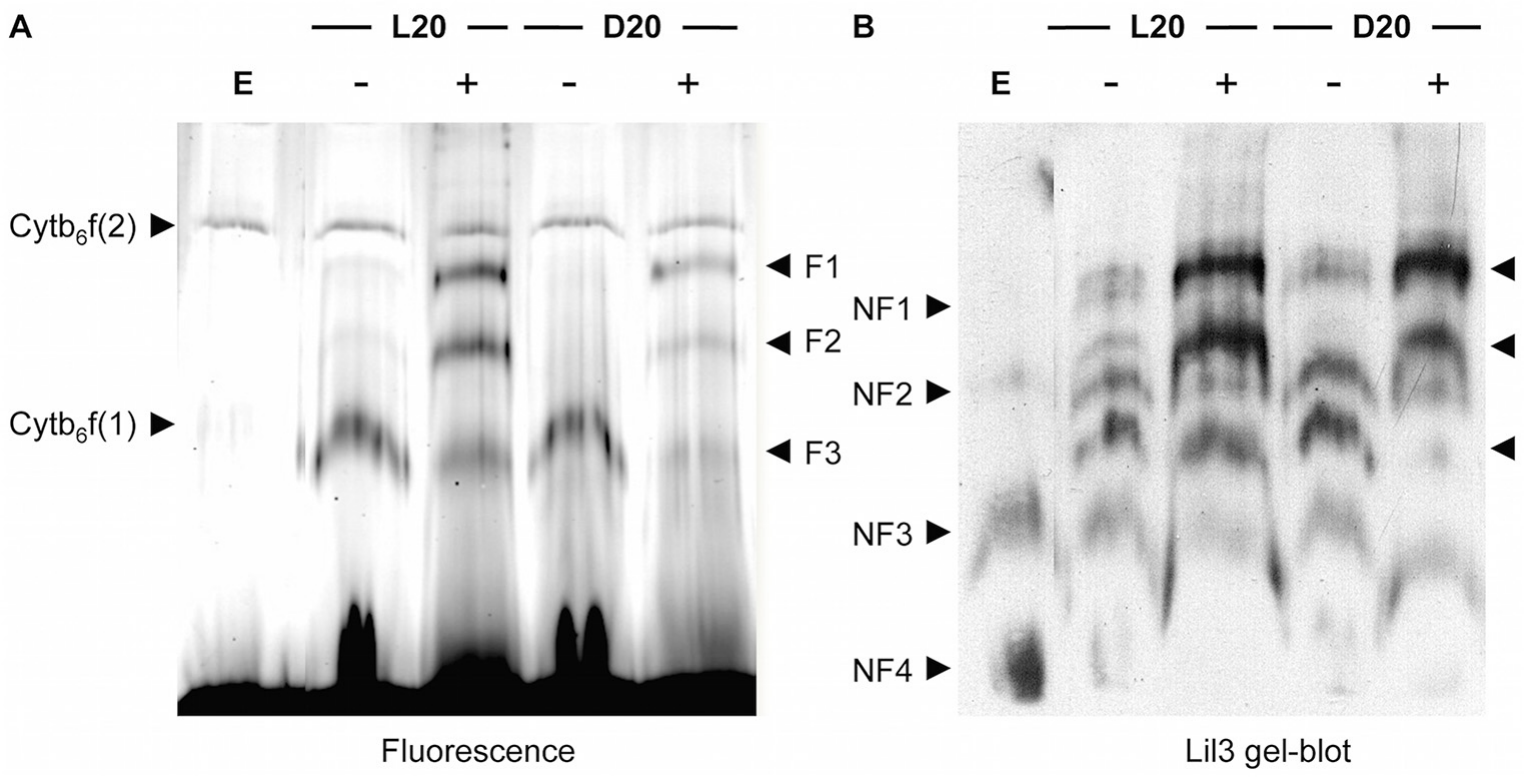
Assembly state changes of Lil3 by Chlorophyllide and Chlorophyll. Assembly of the Lil3 protein was investigated in isolated etioplasts (E). Plastids were incubated in the absence (-) or presence (+) of GGPP and upon illumination (L20) or in darkness and presence of Zn-pheide (D20) for 20 min at 25°C. Membranes were solubilized and protein complexes separated by LN-PAGE in a 7.5% acrylamide gel. Native gels were scanned by laser excitation at λ = 633nm (A, Fluorescence). The Cyt b_6_f band was labeled upon immunological detection of Cyt b_6_ (A, Cyt b_6_f). Lil3 protein in fluorescent bands (F1-3) and Lil3 protein in non-fluorescent bands (NF1-4) was identified using polyclonal antibodies directed against Lil3 (B, Lil3 gel-blot).

Separation of protein complexes from etioplast membranes by native LN-PAGE reveals two fluorescent protein bands and a high fluorescence emission at the gel front (Fig 1, Fluorescence). The two fluorescent protein bands had earlier been identified as the low molecular weight monomeric (Cyt b_6_f (1)) and the high-molecular weight dimeric (Cyt b_6_f (2)) form of the Cyt b_6_f complex [18]. In addition Protochlorophyll was identified in the dimeric Cyt b_6_f (2) band [18]. In 4.5 day-old dark-grown etiolated seedlings, a high concentration of POR, and 80–120 pmol of phototransformable Pchlide had been determined per 10^7^ etioplasts [19]. Interestingly, no Pchlide could be extracted from the fluorescent bands in this work; however, the extraction of Pchlide at the gel front indicated that Pchlide was released from POR during solubilization of the membrane and native PAGE analysis. Illumination of etioplasts and photoreduction of Pchlide resulted in a marked increase in the fluorescence intensity at about the mobility of the Cyt b_6_f (1) band (Fig 1, Fluorescence, F3). Also, two bands of higher molecular weight, but very low fluorescence intensity were detectable (Fig 1, Fluorescence, F1 and F2). Data indicated that the photoreduction of Pchlide resulted in a more stable interaction of Chlide with POR or that additional proteins assembled the newly synthesized Chlide.

In etioplasts isolated from etiolated barley seedlings, Chlide is only effectively esterified to yield Chl when GGPP is supplemented [19]. When GGPP was added, Chlide synthesis and esterification resulted in a strong fluorescent increase in the two higher molecular mass bands, while the Chlide specific fluorescent band was maintained albeit at a reduced intensity (Fig 1, Fluorescence, F1, F2 and F3). This indicated that Chlide triggers accumulation of the F3 band, and that GGPP dependent esterification of Chlide may trigger accumulation of F1 and F2. Whether light or Chlide triggered assembly of the F3 band was tested without GGPP supplementation by addition of Zn-pheophorbide (Zn-pheide) to etioplasts maintained in darkness (Fig 1, Fluorescence, lane 4 and 5). Accumulation of fluorescence in F3 was triggered by Zn-pheide alone, whereas accumulation of fluorescence in F1 and F2 required the presence of Zn-pheide and GGPP. Interestingly, buildup of the F1 and F2 bands correlated with the loss of F3, and most importantly, the correlated reactions did not require a preceding phototransformation. Instead, the presence of Chlide and the esterification of Chlide in darkness were sufficient to trigger the assembly state changes. Data therefore excluded that the increase of fluorescence in the F3 band was based on a binding of Chlide by POR. It was therefore investigated whether other proteins could bind Chlide in the F3 band.

A promising candidate for binding the *de novo* synthesized chlorine molecules was identified by comparison of fluorescence with gel-blot data of the native gel. A peptide specific antibody against the Lil3 protein (S1 Fig) revealed a positive correlation between the signal strength of the Lil3 antibody and the Chlide (F3) and GGPP dependent fluorescence (F1, and F2) (Fig 1, Fluorescence and Lil3 gel-blot). In addition, four Lil3 containing bands were identified in the gel-blot analysis that did not show a corresponding fluorescence in the native gel (Fig 1, Lil3 gel-blot, NF1, NF2, NF3 and NF4). Hence, Lil3 protein assembled in higher molecular weight assembly states already in the absence of chlorines and the assembly state of Lil3 containing bands was changed in the presence of Chlide and GGPP. Lil3 had earlier been shown to bind Chl during deetiolation [17]; however, the structural and functional context for binding Chlide and Chl was unclear. It was therefore investigated whether the enzymes POR, CHS, and GGR interact with Lil3 in the different fluorescent bands and how Lil3 may contribute to a binding of Chlide and Chl.

### POR and CHS are potential interaction partners of Lil3

**Fig 2:**
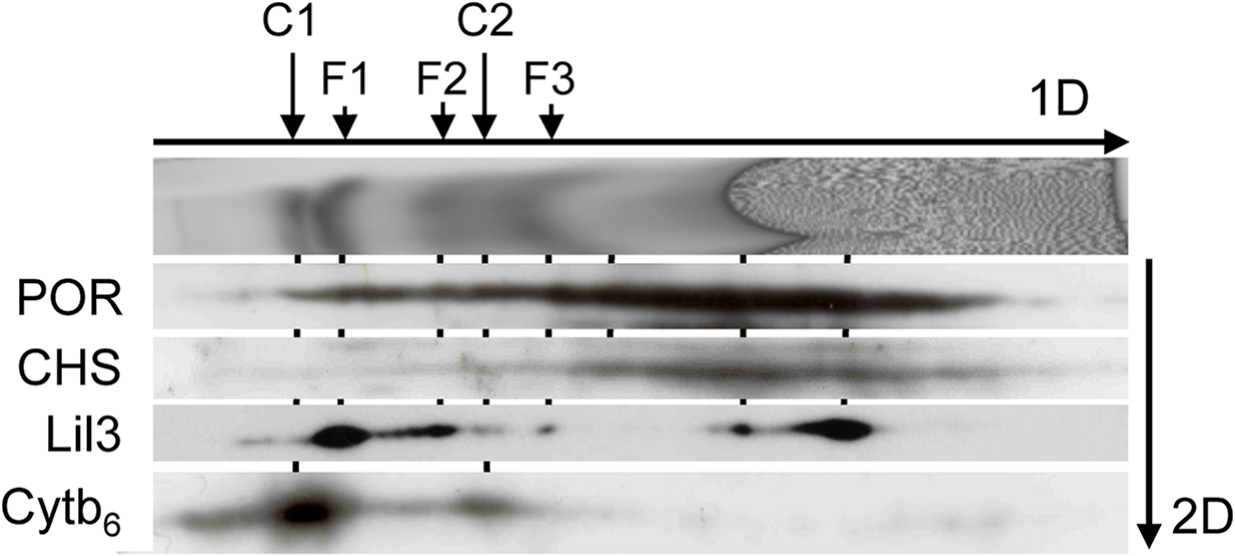
Comigration analysis of Lil3, CHS, POR, and Cyt b_6_ by LN-PAGE. Plastids were isolated from etiolated barely seedlings illuminated for 10 seconds at 25°C. Membranes were solubilized and protein complexes were separated by LN-PAGE (1D). The fluorescence of bands at 670 nm was analyzed by excitation scanning of gels at 633 nm. The mobility of tetrapyrrol binding proteins Lil3, CHS, POR, and Cyt b_6_ was investigated by denaturation of the native gel lane and separation of protein subunits using second dimension SDS-PAGE and gel blot analysis (2D). Mobility of fluorescent Cyt b_6_f bands C1 and C2, and of the Lil3 bands F1, F2 and F3 was labeled according to the antibody signals of the Cyt b_6_ and the Lil3 subunits. Overlapping mobility of proteins in the 1D gel has been connected by a vertical line. A peptide specific antibody against the C-terminus of Lil3 was employed (S1 File).

The protein composition of the fluorescent bands was investigated further by gel-blot analysis upon 2D LN-/SDS-PAGE. Etioplast membranes were isolated from etiolated plants after illumination for 10s. Protein complexes solubilized from the membranes were separated by LN-PAGE and protein subunits in native bands were resolved by SDS-PAGE. The gel-blot of the second dimension gel was sequentially probed with polyclonal antibodies against Lil3, POR, CHS, and Cyt b_6_ (Fig 2 and S2 Fig). Lil3 was identified at about 25 kDa in spots corresponding to native bands F1, F2, F3. In addition, Lil3 was identified in two spots within the molecular weight range of micelles containing free pigment (Fig 2, Lil3). CHS was detected at about 30 kDa (Fig 2, CHS) with highest concentration of the protein corresponding to the two Lil3 spots at the edge of the free pigment and an extension of the signal especially towards the native F3 band (Fig 2, CHS). POR was identified at about 40 kDa (Fig 2, POR). The mobility of POR during native PAGE correlated well with CHS, and with the Lil3 subunits of the native Lil3 bands F1, F2, and F3 and with the two Lil3 bands that were identified at the pigment front of the native gel (Fig 2).

Analysis of the Cyt b_6_ subunit identified the two mobility states of the Cyt b_6_f complex in native PAGE corroborating a dimeric and monomeric assembly state (Fig 2, Cyt b_6_f). Fluorescence analysis after LN-PAGE and gel-blot analysis of the Cyt b_6_ subunit showed furthermore that the mobility of the Cytb_6_f dimer was distinct from the native F1 band (Fig 2, Cyt b_6_f). The Cytb_6_f monomer showed some mobility overlap with a Lil3 signal intermediate between the F2 and F3 bands (Fig 2, Cyt b_6_f). The overlapping mobility of protein subunits Lil3, CHS, and POR indicated an interaction of the three proteins in the fluorescent native PAGE bands; however, it remained unclear whether additional proteins were assembled in the fluorescent bands. Therefore, the gel region of bands F1, F2, and F3 was analyzed by mass spectrometry before and after induction by Chlide and GGPP.

### GGR accumulation in bands F1 and F2 is light induced

**Fig 3:**
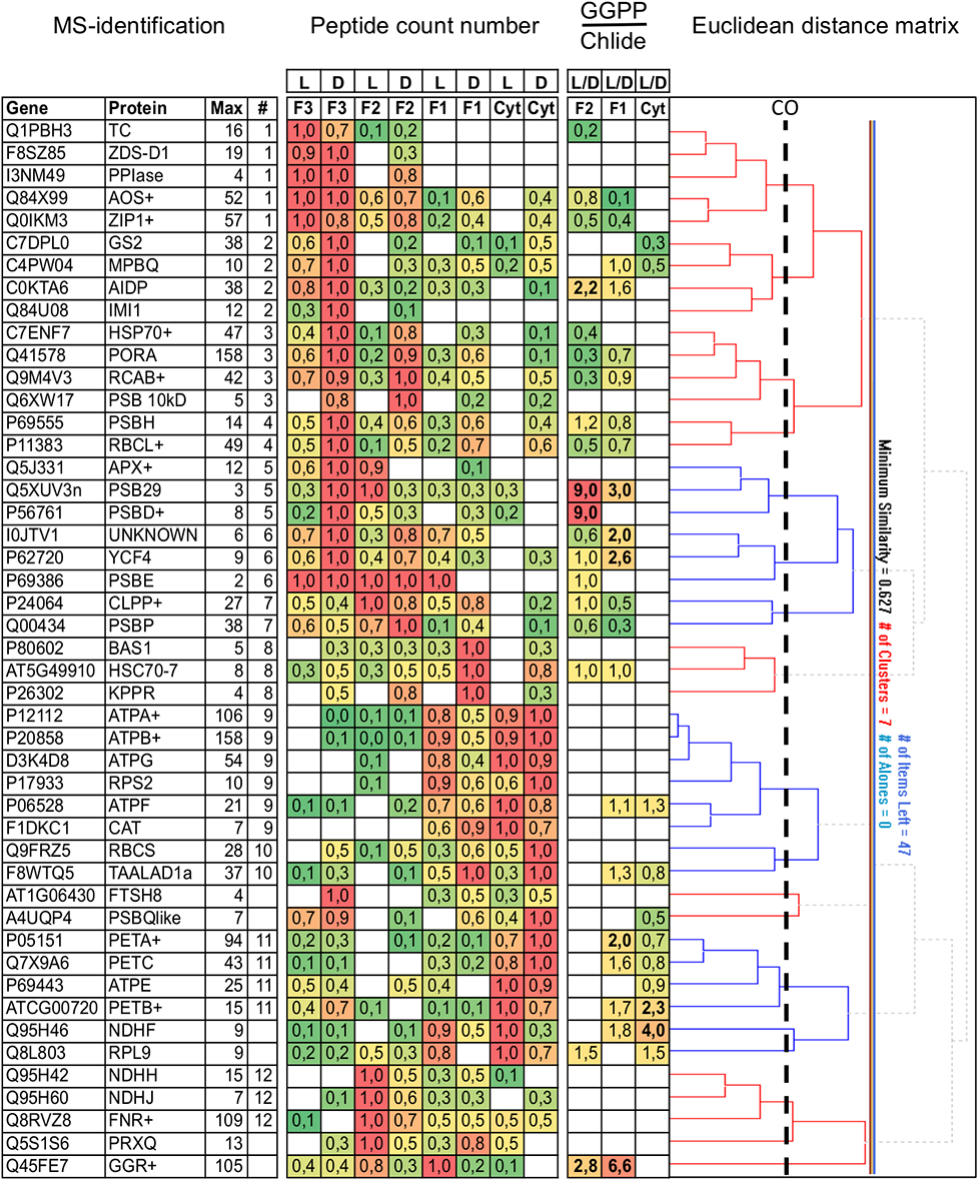
Changes in the protein composition of Lil3 bands. Protein changes in the molecular mass region of fluorescent native PAGE bands labeled Cyt b_6_f, F1, F2, and F3 were investigated by comparative MS and bioinformatics analysis (Methods, S1 Table). Gene annotation (gene), protein name (protein) and maximum peptide count number (max) (MS identification) as well as normalized peptide count numbers are listed for each band. The ratio of changes between the dark (D) and Chlide (L, F3), and Chlide plus GGPP (L, F1 and F2) values are plotted (GGPP/Chlide, L/D) for the Cyt b_6_f (Cyt), the Lil3-F1 (F1) and the Lil3-F2 (F2) band. Gene and band arrangement was mathematically calculated by the position in the Euclidean distance matrix. Cluster numbers (#) were given top down at a minimum similarity cut off (CO) value at 0.766 (dashed line).

A comparative MS experiment was set up to reveal general changes in the protein composition of fluorescent bands between the dark, the Chlide, and the GGPP induced state. Membranes were solubilized and protein complexes separated by native PAGE before and after induction of Lil3 assembly. Fluorescent bands and the corresponding non-fluorescent gel regions in the non-induced states were identified and gel regions excised (S3 Fig). The total peptide count was determined for all identified proteins, and proteins with a peptide cut-off value higher than ten were selected for hierarchial cluster analysis (Fig 3 and S1 Table) [20, 21]. Changes in the protein composition between the induced and the non-induced states and the ratio of protein changes were determined to selectively show which proteins were associated with an increase of fluorescence in the F1 and F2 band relative to a decrease in the F3 band (Fig 3). The band containing the dimeric Cyt b_6_f was used as a control. MS analysis revealed a selective 6-fold light-induced increase of GGR in the F1 and a 3-fold increase in the F2 band, relative to F3 (Fig 3). This showed that GGPP triggered the assembly of GGR in the F1 and F2 bands. In contrast, GGR levels were found constant in the gel region of the F3 band in the absence and presence of Chlide and the enzyme was not detected in the molecular weight region of the Cyt b_6_f complex.

Two protein subunits of photosystem II (PSII) also showed a remarkable increase in bands F1 and F2. A 3-fold induction was found for Psb29 in the F1 band and a 9-fold induction was found for both PsbD and Psb29 in the F2 band. Both proteins clustered with thylakoid based ascorbate peroxidase (APX) (Fig 3, Cluster 5).

In the dimeric Cyt b_6_f band, the comparative MS analysis revealed a 4-fold increase in the plastid encoded NAD(P)H plastoquinone oxidoreductase subunit 5 (NDHF) [22] (Fig 3). However, protein clustering was less stringent for the NDH subunit as compared to the identification of Cyt b_6_f subunits PetA, PetB and PetC in cluster 11 (Fig 3). The protein clusters identified in the native Lil3 and Cyt b_6_f bands reflected complexes for Chl synthesis (Fig 3, clusters 1–3), photosystem assembly (clusters 4–8), ATP synthesis (cluster 9), and electron transport via the Cyt b_6_f (cluster 11) and the NDH complex (cluster 12). Proteins FTSH8, PSBQlike, NDHF, RPL9, PRQX, and GGR did not cluster stringently (Fig 3). The experimentally induced changes in the GGR levels of fluorescent bands F1 and F2 and the gel-blot based indications for interaction between Lil3 and the pigment binding enzymes CHS and POR encouraged us to investigate a direct interaction between the Lil3 protein and CHS, POR, and GGR in a split ubiquitin system and to investigate the pigments bound to the Lil3 protein bands.

### Lil3 and CHS interact in a split ubiquitin Y2H screen

**Fig 4:**
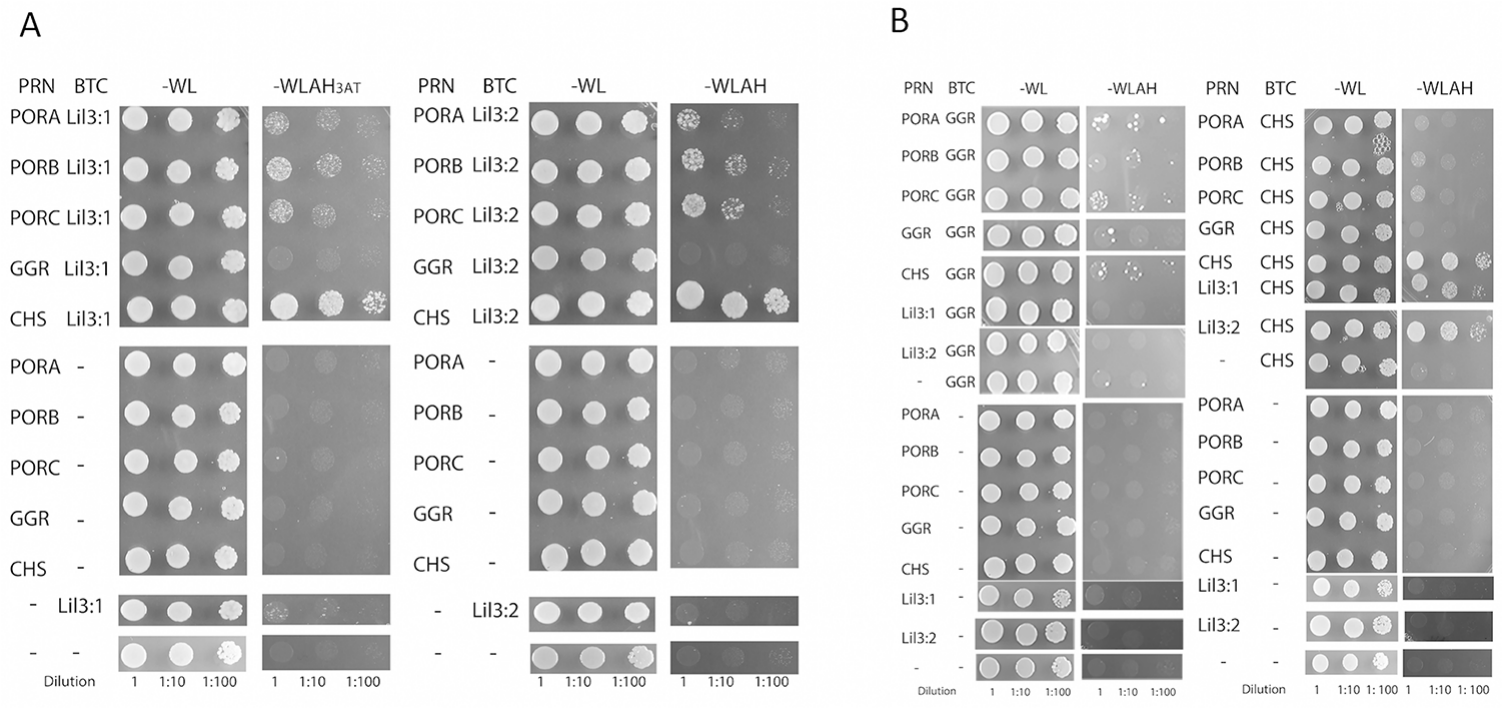
Direct interaction analysis between POR, Lil3, CHS, and GGR. NMY51cells were co-transformed with BTC-*Lil3*:*1* or BTC-*Lil3*:*2* and, PRN-*PORA*, PRN-*PORB*, PRN-*PORC* PRN-*CHS*, PRN*-*GGR (A), with BTC-*GGR or* BTC-*CHS* and, PRN-*PORA*, PRN-*PORB*, PRN-*PORC*, PRN*-GGR*, PRN-*CHS*, PRN-*Lil3*:*1*, PRN-*Lil3*:*2 (B*). Serial dilutions of the yeast strain were made to evaluate the specificity of the interaction.

The direct interaction of Lil3 with the potential binding partners CHS, POR, and GGR was investigated by the split ubiquitin system in yeast. Expression of protein pairs resulting in growth of yeast (+) under suppressing growth conditions was plotted (Fig 4 and S2 Table). Lil3:1 and Lil3:2 were fused to the N-terminus of the Cub moiety protein and to the C-terminus of the NubG moiety protein to generate BTC-*lil3*:*1*, BTC-*lil3*:*2*, PRN*-lil3*:*1* and PRN*-lil3*:*2*, respectively. CHS, PORA, PORB, PORC and GGR were fused to the C-terminus of the NubG moiety protein to generate PRN-*CHS*, PRN-*PORA*, PRN-*PORB*, PRN-*PORC* and PRN*-GGR*. Each of the PRN constructs was co-expressed in yeast NMY51 cells with BTC-*lil3*:*1 and* BTC-*lil3*:*2*. The ability of yeast NMY51 cells to only grow in the absence of histidine by direct protein–protein interaction between the two proteins encoded by the candidate genes was investigated. Among all tested combinations, Lil3:1 and Lil3:2 were found to directly interact with CHS (Fig 4A). Also, a direct interaction between Lil3 and POR was identified (Fig 4A). No interaction was identified between Lil3 and GGR. The lack of interaction between Lil3 and GGR could be due to the orientation of the genes in the expression vector. Therefore, GGR and CHS were fused to the N-terminus of the Cub moiety protein to generate BTC-*GGR* and BTC*-CHS*, respectively. Interactions between CHS and Lil3:1 and Lil3:2 remained positive. CHS also showed an interaction with itself (Fig 4B). GGR was found to interact with POR, CHS, and with itself (Fig 4B). However, CHS did not directly interact with any of the POR proteins or GGR. No direct interaction between GGR and Lil3 could be identified (Fig 4B). An interaction of Lil3 with the two enzymes CHS and POR indicated that Lil3 participates in the regulation of Chlide esterification. It was therefore investigated next whether Lil3 binds Chlide.

### In fluorescent band F3, Chlide, Chl_GG_ and Chl_PY_ accumulate

**Fig 5:**
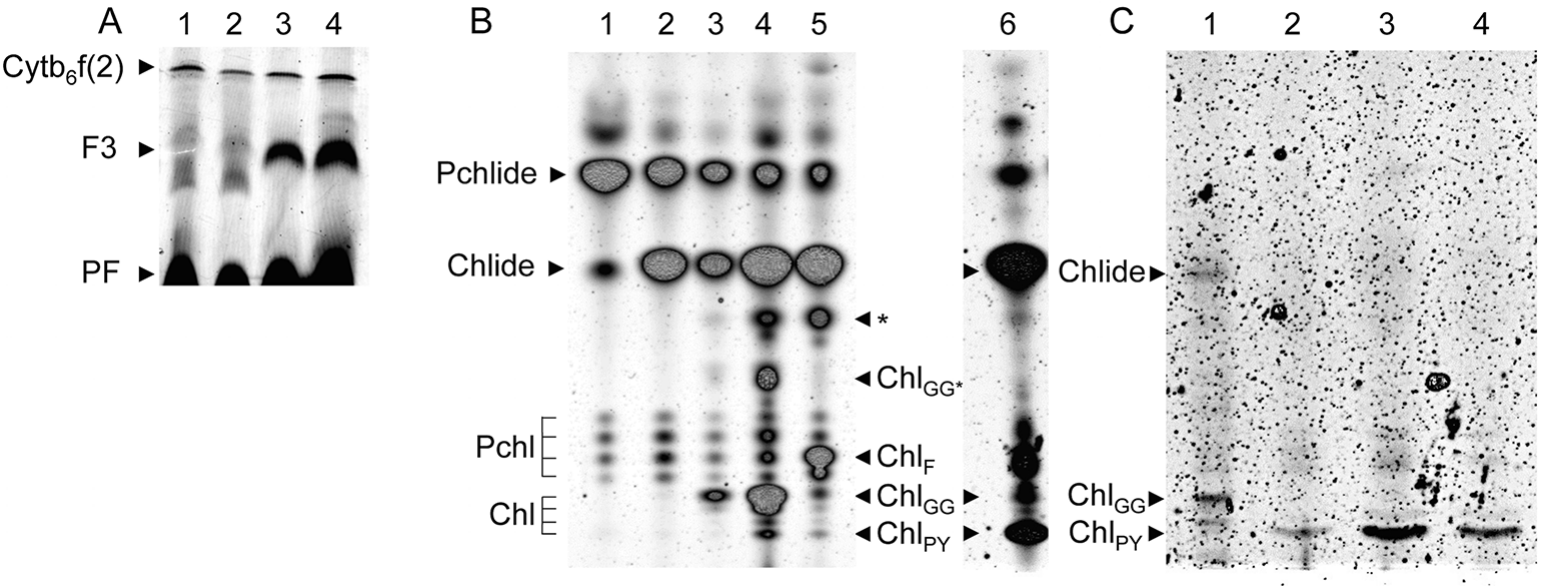
Determination of Chlide, ChlGG, and ChlPY in fluorescent Lil3 bands. For identification of Chlide specific fluorescence in protein complexes, isolated etioplasts (1x10^8^) were incubated without (lanes 1, 3) or supplemented with (lanes 2, 4) GGPP on ice for 1 min in darkness, plastids were frozen on dry ice and either maintained in the dark (lanes 1, 2) or exposed to light for 10 seconds (lanes 3, 4) (A*)*. Plastids were lyzed, membranes were solubilized and protein complexes separated by LN-PAGE (3–12% acrylamide gradient) (A). For identification of pigments bound to fluorescent Lil3 bands, pigments were extracted from etioplasts (B) or from gel-bands after separation by 7.5% native PAGE (C). For identification of pigments in etioplast membranes (B), acetone extraction was conducted using etioplasts kept in darkness (B, lane 1), illuminated (B, lane 2), illuminated and incubated with GGPP (B, lane 3), or illuminated in the presence of GGPP and NADPH (B, lane 4), or FPP and NADPH (B, lane 5). Etioplasts isolated after a 10 sec *in vivo* illumination of etiolated plants (B, lane 6) were extracted and loaded as control. For identification of pigments bound to protein complexes (C), fluorescent Lil3 bands F3 (C, lane 1 and 2), F2 (C, lane 3), and F1 (C, lane 4) were extracted. Pigment synthesis was induced by a 10 s light exposure of etioplasts (C, lane 1) or of etiolated plants (C, lane 2–4). HPTLC and native gels were scanned for fluorescence by laser excitation at λ = 633nm. The position of fluorescent protein complexes Cyt b_6_f, F3, and of pigments in the gel front (PF) of the native gel (A), and of pigments Pchlide, Chlide, Pchl, Chl_GG_, Chl_PY_, Chl_F_, and unidentified GGPP and FPP dependent tetrapyrrol derivatives (Chl_GG*_ and Chl_F*_) after HPTLC separations (B, and C) are marked.

In order to determine whether Lil3 binds Chlide in the F3 band, the changes in pigment composition were investigated in the plastid membrane and in the fluorescent F1, F2, and F3 bands. In order to study Chlide induced changes in the absence of downstream enzymatic processes, etioplasts were frozen on dry ice before illumination. Under these conditions phototransformation of Pchlide is possible, but the reaction speed of subsequent enzymatic steps is decreased [23] (Fig 5). Esterification was controlled by supplementation of etioplasts with GGPP before or after phototransformation. For analysis of pigment accumulation in plastids, frozen plastids were melted into acetone. For analysis of pigments bound to the fluorescent protein bands, membranes were solubilized in darkness by thawing the frozen etioplasts in a solubilization buffer on ice. Pigments were extracted from the gel-bands after native PAGE and separated by HPTLC. Tetrapyrrol derivatives were analyzed by fluorescence scanning (Em 670 BP30, Ex 633 nm) (Fig 5).

The fluorescence emission of Pchl associated with the Cyt b_6_f complex was found unchanged under all conditions (Fig 5A). In addition, no changes in the relative amount of Pchl-GG,-DHGG,-THGG and Pchl-PY were identified after separation of etioplast membrane extracts (Fig 5B, lanes 1–3, Pchl). Illumination of the frozen plastids in the absence and presence of exogenously added GGPP clearly showed that the Lil3 band F3 increased selectively (Fig 5A, lane 3, F3). The corresponding pigment extract from the membranes showed the selective increase in the Chlide signal and indicated that the fluorescence in the native band F3 originated from Chlide binding (Fig 5B, lanes 2 and 3). In the presence of GGPP supplementation the amount of Chlide also increased and in addition, a band corresponding to Chl_GG_ was identified (Fig 5B, lane 3). Supplementation of the etioplasts with GGPP and NADPH increased the yield of the Chl_GG_ band and in addition, a band corresponding to the mobility of Chl_PY_ was detected (Fig 5B, lane 4). The identity of Chl_GG_ in the presence of GGPP supplementation was verified since two more GGPP dependent fluorescence bands were detected (Fig 5B, lanes 4 Chl_GG*_, and *). The identity of the Chl_GG_ and Chl_PY_ bands was corroborated by exchange of the tetraterpenoid GGPP against the triterpenoid farnesyl-pyrophosphate (FPP). In the presence of FPP, Chl_F_ was formed and the Chl_GG_ signal and one of the non-polar GGPP dependent signals (Chl_GG_*) were lost (Fig 5B, lanes 4 and 5, Chl_F_, Chl_GG*_, and *). Finally, the position of Chl_PY_ on the HPTLC plates was verified by separation of etioplast extracts isolated from etiolated plants illuminated for 10s *in vivo* (Fig 5, lane 6, Chl_PY_). Data corroborated that in isolated etioplasts no GGPP was accessible for esterification of the *de novo* synthesized Chlide (Fig 5, lane 2). Also, hardly any Chl_GG_ and GGPP dependent signals were identified when plants were illuminated *in vivo* (Fig 5, lane 6). This indicates that the concentration of GGPP available for esterification in etioplasts is low *in vivo*.

When pigments were extracted from the F3 band after illumination of etioplasts, Chlide and Chl_GG_ were identified (Fig 5C, lane 1). This corroborated that POR and CHS were enzymatically active and indicated that pigments in F3 were bound to the enzymes or to Lil3. When pigments were extracted from the three fluorescent protein bands F1, F2, and F3 upon a 10s illumination of the etiolated plant, Chl_PY_ was identified in all fluorescent bands (Fig 5C, Lane 2, 3 and 4). This corroborated that all three Lil3 containing bands participated in the synthesis of Chl_PY_.

## Discussion

**Fig 6:**
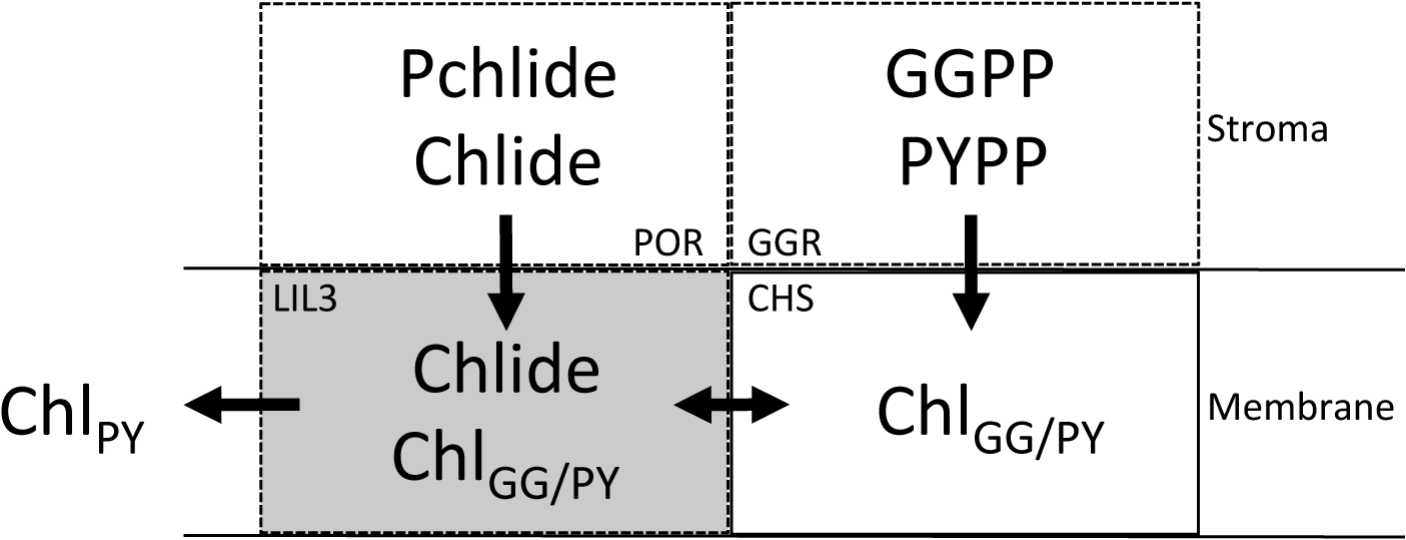
Assembly of proteins POR, Lil3, CHS, and GGR. The enzymes protochlorophyllide oxidoreductase (POR, EC 1.3.1.33), and geranygeranyl reductase (GGR, EC1.3.1.83) are stromal enzymes (Stroma). Chloropohyll synthase (CHS, EC 2.5.1.62), and the light-harvesting like protein Lil3 are membrane integral proteins of the chloroplast (Membrane). All four proteins are proposed to interact in barley etioplasts during synthesis of phytylated Chl. POR binds protochlorophyllide a (Pchlide) and synthesizes chlorophyllide a (Chlide) in the light. Chlide released from POR (arrow) or chemically supplied, assembles with a protein band containing Lil3 and CHS. At low temperature Chlide and Chl_GG_ are isolated from the F3 band (grey background). At room temperature, fluorescence and molecular mass of Lil3 shifts, GGR accumulates in the bands F1, and F2 (Figs 1 and 3) and F1, F2, and F3 bind Chl_PY_ (Fig 5, grey background). The F2 band accumulates psb29 and D2 indicating that Lil3 participates in the delivery of Chl_PY_ (left arrow). POR, GGR, and Lil3 are detected in all fluorescent complexes F1, F2, and F3 by mass spectrometry or gel-blot analysis (dotted boxes). The presence of CHS, and GGR in fluorescent bands is indicated by accumulation of Chl_GG_ and Chl_PY_. Lil3 is shown to directly interact with CHS, and POR in a split ubiquitin based Y2H screen (Fig 4). No direct interaction between GGR and Lil3 or POR and CHS was identified.

Four levels of evidence indicate that Lil3 directly assembles with proteins regulating Chl biosynthesis (Fig 6). The electrophoretic mobility of Lil3 is decreased when etioplasts are supplemented with Chlide (Fig 1). In the absence of GGPP the electrophoretic mobility of Lil3 correlates with the mobility of POR and CHS in native PAGE (Fig 2). Chlide, Chl_GG_, and Chl_PY_ are identified in the F3 band (Fig 5) and Lil3 directly interacts with CHS and POR in a split ubiquitin screen (Fig 4). It is therefore concluded that Lil3 interacts with the two enzymes POR and CHS during synthesis of Chl_PY_ in barley etioplasts.

In the presence of GGPP, Lil3 associates with the two fluorescent protein bands F1 and F2. In both bands, Chl is isolated in the phytylated form (Fig 5). This indicates that Lil3 participates in esterification of Chlide by CHS. It remained open, whether Chlide and Chl_GG_ are bound to Lil3 or to CHS. Lil3 binding of Chlide in the fluorescent band F3 could mediate exposure of the carbonyl group thereby allowing esterification by CHS. The lack of Chl_GG_ or Chl_PY_ accumulation in isolated etioplasts after illumination, and the sole accumulation of phytylated Chl in the F1, F2, and F3 bands *in vivo* (Fig 5) indicated that GGR provides CHS with PYPP in etiolated plants *in vivo*. Lil3 binding of Chl_GG_ after *in vitro* supplementation with GGPP and of Chl_PY_ *in vivo* could have a function in exposing the esterified chlorine for photosystem assembly.

In *Synechocystis* PCC6803, the one-helix Lil-homolog HliD, Ycf39, YidC, and CHS were shown to interact [16], and a coregulation of Chl synthesis and membrane integration of photosystem proteins was concluded from a copurification of CHS and unassembled D1 with a Ycf39-Hlip complex [24]. Interestingly, GGR was not isolated with the complexes regulating Chl synthesis in *Synechocystis* PCC6803 [16, 24]. The enzyme also did not cluster with any of the proteins identified in the fluorescent Lil3 bands in our bioinformatic analysis of the MS datasets (Fig 3). The GGPP dependent accumulation of GGR in the fluorescent bands F1 and F2 may result from assembly with proteins that participate in the regulation of GGPP reduction but that were not identified in the MS approach or were excluded from the hierarchial cluster analysis due to the selected cut-off value (Fig 3). Lil3 was identified in the F1 and F3 bands, but count numbers were below the cut-off value and data were excluded from further analysis. Loss of GGR has been reported as an explanation for the loss of Chl_PY_ and tocopherol in a Lil3 double knockout line of Arabidopsis thaliana [11]. However, the low-growth phenotype and low yield of Chl_GG_ remained unexplained [11]. GGR mutants (ΔchlP) in Synechocystis PCC6803 demonstrated that accumulation of Chl_GG_ and growth of mutants were comparable to WT in low light [25]. According to our model, the absence of Lil3 may lead to a low yield of Chlide esterification and hereby limit autotrophic growth. The loss of GGR in the mutant lines may be explained by the loss of the direct interaction between Lil3 and CHS as shown here. This interaction may provide the structural basis to stabilize GGR against degradation in Arabidopsis thaliana.

### Interaction of Lil3 with CHS

Characterization of a complex containing CHS and the high-light-inducible Lil homolog HliD in Synechocystis PCC6803 led to the conclusion that HliD is a non-essential component for the function of CHS [16]. Since deletion of HliD increased the Chlide content 6-fold in the deletion strain, the authors concluded that HliD could be responsible for directing the assembly of proteins like Ycf39 into a complex with CHS [16]. As shown here, Lil3 assembles with POR and CHS in barley etioplasts. Phototransformation of etioplasts at low temperature results in accumulation of Chlide and Chl_GG_ in the Lil3 containing F3 band (Fig 5). In the presence of GGPP *in organello*, or illumination of plants *in vivo*, the F3 band is shifted into band F1 and F2 in which GGR and Chl_PY_ accumulate (Figs 1, 3 and 5). This indicates that a complex composed of POR, Lil3, and CHS precedes an assembly of increasing amounts of GGR (Figs 5 and 6).

In the split ubiquitin screen, Lil3 shows direct interaction with CHS and with POR. No direct interaction was found between Lil3 and GGR and between POR and CHS. Further, CHS and also POR were found to directly interact with GGR (Figs 4 and 6). These data indicate that Lil3 and GGR co-assemble in etioplasts with POR and CHS in a substrate dependent manner without interacting directly (Figs 5 and 6). The transfer of Chlide from POR to CHS could require transient binding of Chlide to Lil3 for optimized substrate interaction with CHS and fast reloading of POR. High concentrations of POR accumulate at the membrane periphery in higher plant etioplasts [26]. In order to reload POR, Chlide removal and translocation from POR to the membrane integral CHS is required for rapid accumulation of Chl_PY_ [27–29].

Interestingly, the rate of Chl accumulation is drastically increased in illuminated etioplasts supplemented with GGPP relative to illuminated plants [30]. Our finding that the availability of GGPP both limits Chl_PY_ accumulation and assembly of Lil3-bands F1 and F2 from the Chlide specific Lil3-F3 band, therefore emphasizes the core position of the membrane integral Lil3 to functionally link the three enzymes. Lil3 directly interacts with CHS and POR and may indirectly interact with GGR via CHS and POR (Fig 6). A lack of direct interaction between Lil3 and GGR has recently also been discussed in rice mutants deficient in GGR [31]. Therefore GGR could interact with Lil3 via CHS. The LHC motif in Lil3 could provide the basis for assembly of Lil3 with CHS. The LHC motif binds Chl in the light-harvesting complex (LHC) [32]. The LHC motif in Lil3 could also function for binding Chlide and Chl. We show direct interaction of CHS with both Lil3 and GGR (Fig 4). Chlorine binding to Lil3 could therefore enable the assembly of a ternary Lil3-Chlide/Chl-CHS complex that anchors GGR to the thylakoid membrane for efficient esterification of Chlide with PYPP (Fig 6).

### Structural changes induced by prenylation of Chlide

A comparative MS analysis of the GGPP relative to the Chlide induced changes, revealed a 6- and 3-fold normalized change for GGR in native gel bands F1 and F2, respectively. Furthermore, PSII components PsbD and Psb29 increased 9-fold in Lil3-F2 relative to Lil3-F3 (Fig 3). PsbD is one of two reaction center proteins of PSII and present in the first assembly intermediate during reaction center assembly [33–35]. The psb29 protein was conserved throughout evolution of oxygenic photosynthetic organisms. A plant psb29 mutant showed slow growth, variegated leaves, and light sensitive PSII activity associated with an uncoupling of the proximal antennae indicating a function of psb29 in the biogenesis of PSII [36]. Our finding indicates that accumulation of Chl_PY_ in the F2 band reflects an assembly of Lil3 with single proteins or with a complex composed of at least psbD, and psb29 (Fig 3).

## Materials and Methods

### Plastid isolation and pigment synthesis

Barley seeds (*Hordeum vulgare L*. *cultivar*. *Steffi*, Saatzucht Ackermann & Co, Irlbach, Germany) were grown for 4.5 days at 23°C. Intact etioplasts were isolated and the concentration of plastids determined by counting [19, 34]. For phototransformation of Pchlide, and synthesis of Chlide and Chl, etioplasts were preincubated in the absence or presence of GGPP (1,35 mM, Sigma-Aldrich, St. Louis, USA). For esterification of GGPP (1,35 mM) and Farnesyl pyrophosphate (FPP) (1,3 mM) (Sigma-Aldrich, St. Louis, USA), etioplasts were investigated in the presence of NADPH (0,5 mM Sigma-Aldrich, St. Louis, USA) for 1 min on ice. Etioplasts were transferred to dry ice and either maintained in the dark (D) or illuminated (L) for 2 s with white light via a light guide (10 mEm^-2^s^-1^, Schott, DE). For comparison of Chl synthesis in light and darkness, etioplast were illuminated with white light (50 E/m^2^s) or supplemented with 0,77 nmol of Zn-pheophorbide *a* (Zn-pheide) in darkness in the presence or absence of GGPP for 20 min at 25°C. Pigment concentrations were determined according to Helfrich [37].\

### Native page

A defined amount of etioplasts (10^8^) were lysed and washed two times in TMK and solubilized in a detergent mix composed of 0.38% (w/v) *n*-dodecyl-β-d-maltoside, 0.64% (w/v) digitonin and 0.006% (w/v) lithium dodecyl sulfate (LDS), as described [17]. All steps were performed on ice and non-soluble material was separated by centrifugation (4°C, 16100 rcf, 10 min). Soluble protein extracts were separated by LDS-Native (LN) PAGE on 7.5% (w/w) [17] or 3–12% polyacrylamide gels (Novex, Life technologies, California USA). The cathode buffer was supplemented with 74 μM LDS and pigment binding proteins were detected by fluorescence scanning (filter 670 BP30) using a HeNe laser excitation at 633 nm in a Typhoon scanner (GE Healthcare, Buckingham, GB).

### 2D polyacrylamide gel electrophoresis

Protein complexes separated in bands by LN-PAGE were recorded by fluorescence excitation scanning of the native gel at 633 nm. Fluorescent bands were cut from the native PAGE lanes of each condition (etioplast, light-GGPP and light + GGPP) solubilized and separated in the second dimension [18, 38, 39] Gels were transferred to nitrocellulose membranes [40] and proteins immuno-detected with polyclonal antibodies against CHS (Anti-CWY15 IgY (1:2000), Agrisera, Vännäs, Sweden (2010-01-13), produced in Hen), POR (Anti-POR (PCOR) (1:2000), Agrisera, Vännäs, Sweden, produced in Rabbit), Lil3 (Anti-Lil3 (1:7500), synthetic peptide CQSTWQDDSTSGPKK Agrisera, Vännäs, Sweden, produced in Rabbit), and Cyt b_6_ (Anti-Cyt b_6_ (N-term) (1:10000), Agrisera, Vännäs, Sweden (AS03-034), produced in rabbit).

### Mass spectrometry analysis

For identification of peptides from LN-PAGE bands A-H, digests were analyzed in an Orbitrap Velos mass analyzer, at a resolution of 30000. Data dependent analysis (Top 20) was employed and ions with charge states of 2+ and above were selected for fragmentation. Data was processed using Protein Discoverer (version 1.2.ThermoFisher) and the Mascot search algorithm (Matrix Science, London UK) and were searched against the TAIR10 and Uniprot Wheat database on Mar. 14, and Jan.13, respectively, using a fixed modification of carbamidomethyl (C) and a variable modification of oxidation (M).

### Peptide data processing

Mascot search results were exported in CSV format and the number of peptide-spectrum matches for each identified protein was determined per LN-PAGE band and variable peptide count numbers per protein compiled in a matrix. Total count number was determined per peptide. All values were standardized and clustered row by row, and column by column using complete linkage in Hierarchical Cluster Explorer 3.5 (HCE3.5) (http://www.cs.umd.edu/hcil/hce/) [20, 21]. A Euclidean distance matrix was calculated for all proteins identified by a minimum of 10 counts. Clustering results were analyzed in the form of a heat map and two dendograms, showing clustering of proteins (rows), and bands (columns). Standardized and clustered datasets were analyzed using OriginLab 9.0 (OriginLab Corp., Northampton, USA) and data normalized (0/1) per row. For annotation of protein assembly, and annotation of untagged proteins, dendrograms were cut at an euclidean distance of 0.6. Normalized peptide differences (Chlide induced minus dark state versus Chl induced minus dark state) and ratios (Chlide induced/Dark state versus GGPP induced/Light state) of corresponding bands were determined.

### Thin layer chromatography

For pigment extraction, etioplasts frozen on dry ice were thawed into an organic phase composed of 80% (v/v) acetone, 20% (v/v) water on ice. Organic phase extracts were stored overnight (- 20°C). For separation, pigment extracts were centrifuged (4°C, 20800 rcf, 10 min) and supernatants loaded on reversed phase (C18) high-pressure thin-layer chromatography (HPTLC) plates (Merck, Darmstadt, DE) and plates developed in a solvent composed of 58.8% (v/v) acetone, 39.2% (v/v) methanol, 2% (v/v) water. From native complexes, pigments were extracted utilizing OMX-S (Soliden, Seefeld-Hechendorf, Germany) after tryptic digestion of proteins (www.omx.de) and pigments concentrated by a C18 stagetip (Thermo Fisher Scientific Inc., Rockford, USA) preactivated by 80% (v/v) acetonitrile and eluted in 80% (v/v) acetone, 20% (v/v) 50 mM Hepes (pH 8.0). HPTLC plates were scanned for fluorescence emission (excitation 633 nm/670 BP30 emission filter) in a Typhoon scanner (GE Healthcare, Buckingham, GB).

### Split Ubiquitin Assay

The split Ubiquitin assay was performed according to the DUALmembrane starter kit (Dualsystems Biotech Inc. Schlieren, Switzerland). Genes were amplified from cDNA using *Pwo* polymerase (Roche, Basel, Switzerland), and cloned into pPCR-Script (Stratagene, California, USA). The coding sequence of *Lil3*.*1* (At4g17600) and *Lil3*.*2* (At5g47110), were cloned into the bait (pBT3-C) and prey (pPR3-N and pPR3C) vectors (Dualsystems Biotech AG, Schlieren, Switzerland). The coding sequences of *ChlP* ((GGR) At1g74470), *ChlG* ((CHS) At3g51820), *PORA* (At5g54190), *PORB* (At4g27440), and *PORC* (At1g03630) were cloned into the pray vector pPR3-N (Dualsystems Biotech AG, Schlieren, Switzerland). In order to verify the interaction, the coding sequences of *ChlP* and *ChlG* were also cloned in the bait vector (pBT3-C). Yeast NMY51 cells were co-transformed with the resulting plasmids according to the manufacturer’s instructions (Dualsystems Biotech AG, Schlieren, Switzerland). In the split ubiquitin assay with Lil3:1 as bait, the selective media (SD–WLAH) was supplied with 5mM *of 3-aminotriazole (3-AT)*. Coexpression analysis experiments were repeated four times, two times in each direction.

## Supporting Information

S1 File Verification of Lil3 antibody specificity. (DOCX)

S1 Fig Specificity of an antibody directed against a Lil3 peptide. Etioplasts were isolated from etiolated barley seedlings illuminated for 10 s with white light. Solubilized membrane extracts corresponding to 1x10^8^ plastids were used. For immunoprecipitation, an antibody directed against the Lil3 peptide QSTWQDDSTSGPKK (C, bold) was employed. Protein precipitates were concentrated by centrifugation and denatured proteins separated by SDS-PAGE (A and B). Proteins were detected by scanning for Cy2 labeling (A) and for secondary antibody specific luminescence in gel blots (B). Solubilized membrane extracts were incubated with Lil3 antibody, Sepharose and detergent (lane 2). In the controls, immunoprecipitation was conducted in assays containing Sepharose, Lil3 antibody, and detergent, but no solubilized membrane (lane 1), Sepharose, solubilized membrane extracts corresponding to 1x10^8^ plastids, but no antibody (lane 3), and only solubilized membrane extracts corresponding to 1x10^8^ plastids, but no sepharose or antibody (lane 4). Peptides were generated from the immunoreactive band and identified by *de novo* sequence analysis (D, 982.47 (2+) peptide) from peptides in the plus 2 charge state (2+) with m/z values of 485.76, 617.33, and 982.47. Identified amino acid sequences were plotted against the Lil3 protein sequence (C, underline) from Hordeum vulgare.
(TIF)

S2 Fig The assembly state of LIL3, CHS, POR, and Cyt b_6_. Etiolated barely seedlings were illuminated for 10 seconds at 25°C. Plastids were isolated and protein complexes were separated by LN-PAGE (7.5% acrylamide). The mobility position of Lil3 and Cyt b_6_f bands separated by native PAGE, are labeled F1, F2, F3 and C1, C2 respectively referring to fluorescent bands determined after native PAGE. Proteins identified in the 2D LN/SDS-PAGE gel-blot by immuno-detection with polyclonal antibodies against Lil3, CHS, POR and Cyt b_6_ are labeled. The molecular mass of marker proteins for the second dimension gel is given in kDa (kD).
(TIF)

S3 Fig Native PAGE band selection and determination of changes in protein composition. Protein bands corresponding to the molecular mass regions of the dimeric Cyt b_6_f (A/E) and the fluorescent Lil3 complexes F1 (B/F), F2 (C/G), and F3 (D/H) were cut from the lanes containing the non-induced (A,B,C,D) and induced (E,F,G,H) states for Chlide (- GGPP) and Chl synthesis (+GGPP) upon LN-PAGE (Fig 1) (boxed bands). Changes in protein composition were identified from etioplast membranes (D) or etioplasts incubated in the light for 20 min by mass spectrometry upon tryptic digestion of bands.
(TIF)

S1 Table Proteins identified in native PAGE bands A-H by mass spectrometry. (DOCX)

S2 Table Interaction analysis of POR, CHS, Lil3, and GGR. (DOCX)
