# Supplementary material for: Lil3 Assembles with Proteins Regulating Chlorophyll Synthesis in Barley
Source: PLoS One. 2015 Jul 14;10(7):e0133145. doi: 10.1371/journal.pone.0133145 (PMC4501709; doi:10.1371/journal.pone.0133145)
Supplement: S1 File — (DOCX) [file pone.0133145.s001.docx]

**Verification of Lil3 antibody specificity**

**Method**

Membranes were isolated and solubilized as described previously, and proteins were labeled with 0.01 mM Cy2Dye Cy^TM^ 2 minimal dye (GE Healthcare, Buckingham, GB). For immunoprecipitation, the antibody directed against the Lil3 peptide QSTWQDDSTSGPKK [1] was coupled to Protein A Sepharose ^TM^ CL-4B (GE-Healthcare Bio Sciences AB, Uppsala, Sweden). Sepharose A (125 mg) was washed and hydrated in 100 µl IP2 buffer (50 mM Tris-HCL (pH 7.5), 150 mM NaCl, 2 mM EDTA). An aliquot of Sepharose A (2.375 mg) was incubated with a detergent mix (2.86 µl) and Lil3 peptide antibody (2 μg) on a rotary wheel (RT, 30 minutes) prior to concentration by centrifugation (1000 rcf, 30 sec, 4°C). The antibody-linked sepharose A beads were washed three times in 200 µl IP2 buffer and then mixed with solubilized membrane extracts (1x10^8^ plastids) for 30 min at 4 °C. After incubation, the mixture was washed with 500 µl IP2 buffer and sepharose and concentrated by centrifugation. Proteins bound to sepharose-antibody beads were released by addition of 20 µl TMK buffer pH 6.8 (10 mM Tris-HCl pH 6.8, 10 mM MgCl_2_, and 20 mM KCl) and 10 µl of preheated (2 min, 72°C) solubilisation buffer (208 mM SDS, 876 mM Sucrose, 1.49 mM bromphenol blue, 200 mM Sodium Carbonate, 200 mM DTT) followed by a 10 min RT incubation. The supernatant was removed from the sepharose mixture by centrifugation (1000 rcf, 30 sec, 4°C) and 18 µl was loaded for separation of immunoprecipitated proteins by SDS-PAGE. Lil3 protein was identified by *de novo* sequence analysis from peptides with charge state 2+ with m/z values of 485.76, 617.33, and 982.47. Identified amino acid sequences were plotted against the Lil3 protein sequence from *Hordeum vulgare* (accession number F2CYY3) [2]

**Result**

The specificity of the antibody was verified by immunoprecipitation and MS analysis (Figure S1). For localization of immunoreactive proteins, etioplast membranes were labeled with Cy2 and the CyDye labelled membrane proteins were immunoprecipitated upon solubilisation of the membrane. Immunoprecipitation resulted in three distinct fluorescent bands (Figure S1 A, lane 2). In contrast, very weak or no fluorescent signals were detected for the controls if membranes, sepharose, or Lil3-antibody were not included in the assays (Figure S1 A, lane 1, 3 and 4). The immunoprecipitated Lil3 was identified by gel-blot analysis. A Lil3 peptide antibody showed one clear immuno-reactive band at about 30 kD and a number of high molecular weight bands (Figure S1 B lane 2). The 30 kD band was missing in the control lacking the membrane extract, but the high molecular immuno-reactive bands were collected indicating to represent antibody and antibody-sepharose aggregates (Figure S1 B, Lane 1 and 2). The Lil3 band showed the same molecular weight as the strongest Cy2 labelled band (Figure S1 A and B, Lane 2). The Cy2 labelled band was cut from the gel and peptides with m/z values of 485.76, 617.33, and 982.47 (D, 982.47 (2+) peptide) in the plus 2 charge state (2+) were generated and identified by *de novo* sequence analysis (Figure S1, C and D) Data show that the Lil3 peptide antibody is able to selectively pull down Lil3 from *in vivo* illuminated plants. The antibody therefore is specific for identification of Lil3 in membrane protein extracts from barley.

1. Reisinger V, Ploscher M, Eichacker LA. Lil3 assembles as chlorophyll-binding protein complex during deetiolation. Febs Letters. 2008;582(10):1547-51. doi: 10.1016/j.febslet.2008.03.042. PubMed PMID: WOS:000258005200026.

2. Matsumoto T, Tanaka T, Sakai H, Amano N, Kanamori H, Kurita K, et al. Comprehensive sequence analysis of 24,783 barley full-length cDNAs derived from 12 clone libraries. Plant Physiol. 2011;156(1):20-8. Epub 2011/03/19. doi: 10.1104/pp.110.171579. PubMed PMID: 21415278; PubMed Central PMCID: PMCPmc3091036.
