# Supplementary figures and images for: Lil3 Assembles with Proteins Regulating Chlorophyll Synthesis in Barley
Source: PLoS One. 2015 Jul 14;10(7):e0133145. doi: 10.1371/journal.pone.0133145 (PMC4501709; doi:10.1371/journal.pone.0133145)

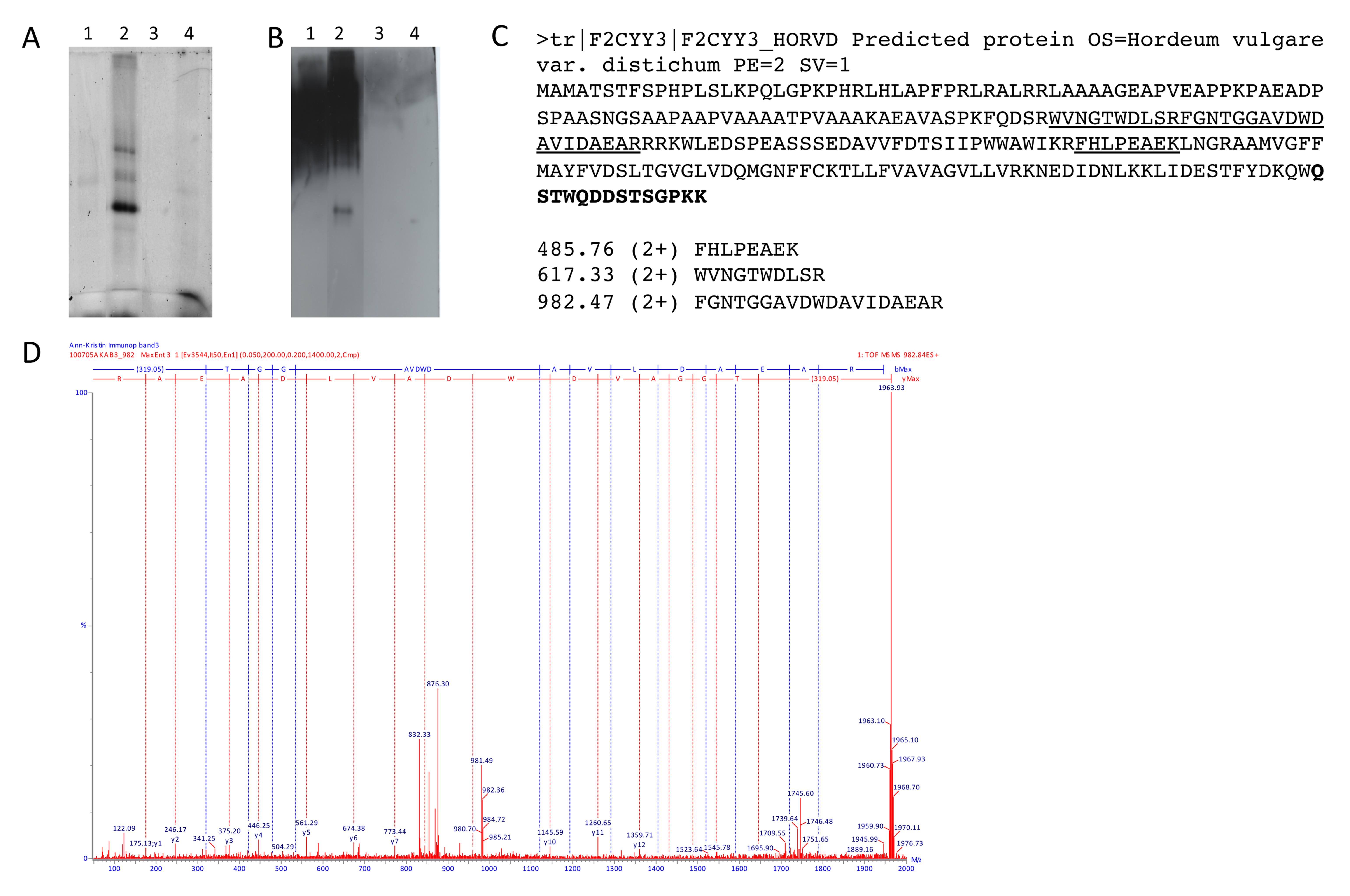

Supplement: S1 Fig — Etioplasts were isolated from etiolated barley seedlings illuminated for 10 s with white light. Solubilized membrane extracts corresponding to 1x10^8^ plastids were used. For immunoprecipitation, an antibody directed against the Lil3 peptide QSTWQDDSTSGPKK (C, bold) was employed. Protein precipitates were concentrated by centrifugation and denatured proteins separated by SDS-PAGE (A and B). Proteins were detected by scanning for Cy2 labeling (A) and for secondary antibody specific luminescence in gel blots (B). Solubilized membrane extracts were incubated with Lil3 antibody, Sepharose and detergent (lane 2). In the controls, immunoprecipitation was conducted in assays containing Sepharose, Lil3 antibody, and detergent, but no solubilized membrane (lane 1), Sepharose, solubilized membrane extracts corresponding to 1x10^8^ plastids, but no antibody (lane 3), and only solubilized membrane extracts corresponding to 1x10^8^ plastids, but no sepharose or antibody (lane 4). Peptides were generated from the immunoreactive band and identified by de novo sequence analysis (D, 982.47 (2+) peptide) from peptides in the plus 2 charge state (2+) with m/z values of 485.76, 617.33, and 982.47. Identified amino acid sequences were plotted against the Lil3 protein sequence (C, underline) from Hordeum vulgare. (TIF) [file pone.0133145.s002.tif]

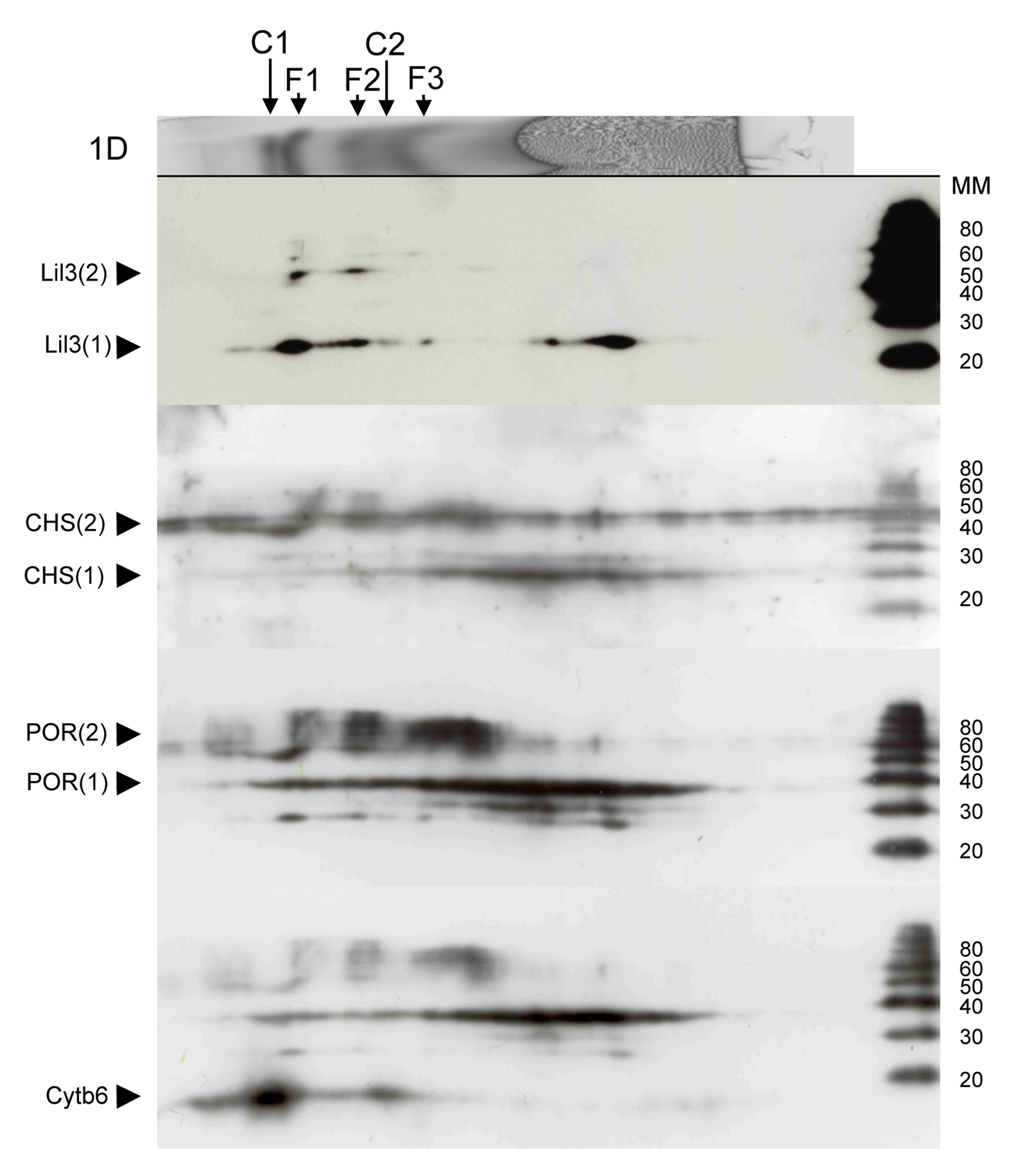

Supplement: S2 Fig — Etiolated barely seedlings were illuminated for 10 seconds at 25°C. Plastids were isolated and protein complexes were separated by LN-PAGE (7.5% acrylamide). The mobility position of Lil3 and Cyt b6f bands separated by native PAGE, are labeled F1, F2, F3 and C1, C2 respectively referring to fluorescent bands determined after native PAGE. Proteins identified in the 2D LN/SDS-PAGE gel-blot by immuno-detection with polyclonal antibodies against Lil3, CHS, POR and Cyt b6 are labeled. The molecular mass of marker proteins for the second dimension gel is given in kDa (kD). (TIF) [file pone.0133145.s003.tif]

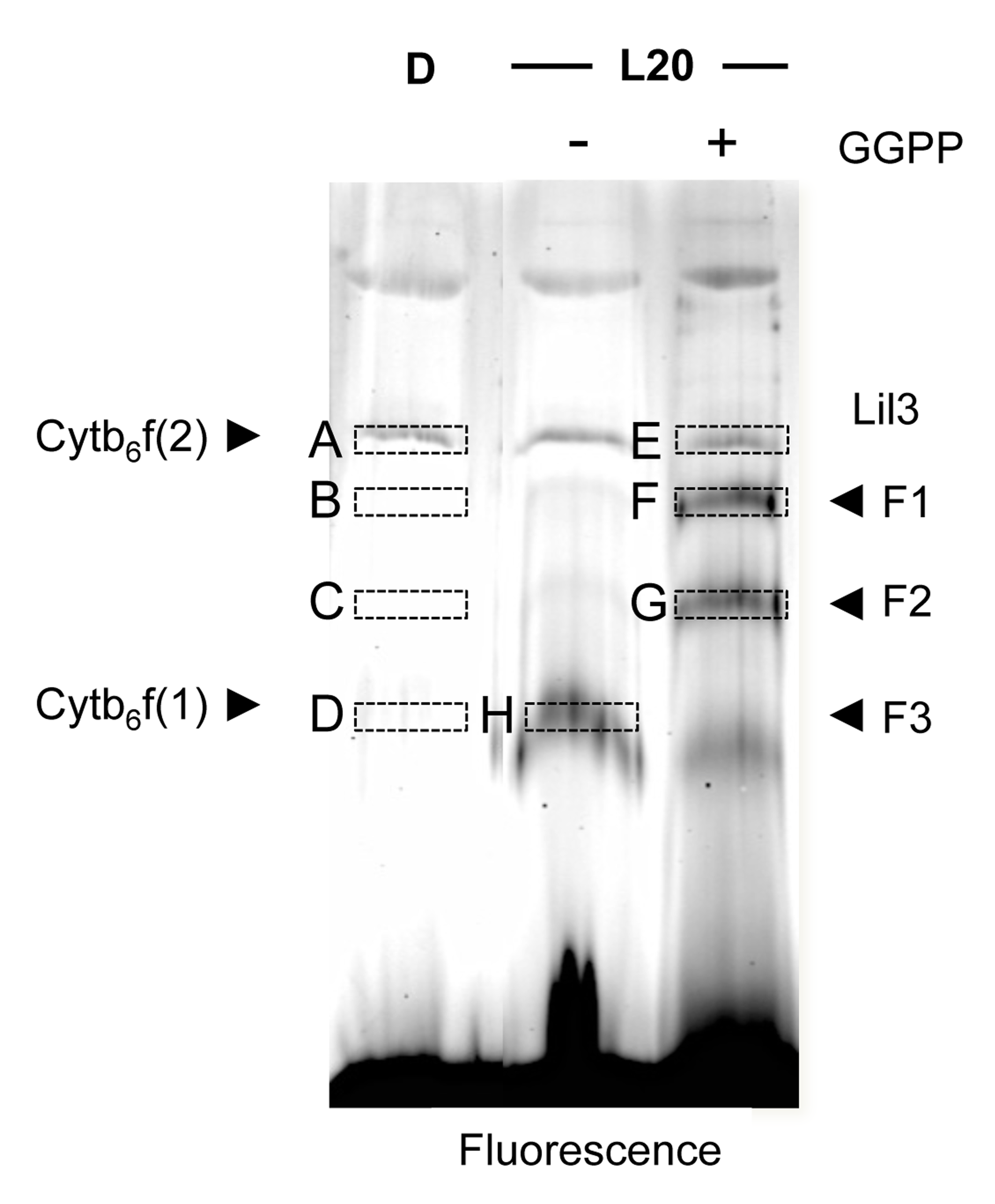

Supplement: S3 Fig — Protein bands corresponding to the molecular mass regions of the dimeric Cyt b6f (A/E) and the fluorescent Lil3 complexes F1 (B/F), F2 (C/G), and F3 (D/H) were cut from the lanes containing the non-induced (A,B,C,D) and induced (E,F,G,H) states for Chlide (- GGPP) and Chl synthesis (+GGPP) upon LN-PAGE (Fig 1) (boxed bands). Changes in protein composition were identified from etioplast membranes (D) or etioplasts incubated in the light for 20 min by mass spectrometry upon tryptic digestion of bands. (TIF) [file pone.0133145.s004.tif]
