# Supplementary material for: Lil3 Assembles with Proteins Regulating Chlorophyll Synthesis in Barley
Source: PLoS One. 2015 Jul 14;10(7):e0133145. doi: 10.1371/journal.pone.0133145 (PMC4501709; doi:10.1371/journal.pone.0133145)
Supplement: S1 Table — (DOCX) [file pone.0133145.s005.docx]

**Table S1. Proteins identified in native PAGE bands A-H by mass spectrometry**

Proteins were identified from tryptic digests of native PAGE bands A-H using Mascot search algorithms and a minimum cut-off of value of 20 (Methods). For each identified protein, the numbers of peptide spectral matches were determined in each of the analyzed fractions compiled in a matrix. Genes corresponding to the same protein are shown with the gene identifier for the protein with the highest count number (+). For each identified protein, the number of spectral counts across the analyzed fractions was normalized to a total of one. A Euclidean distance matrix was calculated for all proteins identified with a minimum of 10 counts. Clusters with a minimum similarity cut off < 0.766 are labeled. The uniprot database (www.uniprot.org) was screened to annotate the proteins function.

| **Cluster** | **Gene** | **Protein** | **Name** | **Function** | **Reference** |
| --- | --- | --- | --- | --- | --- |
| 1 | Q1PBH3 | TC | Tocopherol cyclase | Tocopherol biosynthesis | [1] |
| 1 | F8SZ85 | ZDS-D1 | Carotene 7,8-desaturase | Carotenoid biosynthesis | [2] |
| 1 | I3NM49 | PPIase | Peptidyl-prolyl cis-trans isomerase | Rotamase | [3] |
| 1 | Q84X99 | AOS+ | Allene oxide synthase | Jasmonic acid biosynthesis | [4] |
| 1 | Q0IKM3 | ZIP1+ | Putative leucine zipper protein | Chl biosynthesis | [5] |
| 2 | C7DPL0 | GS2 | Glutamine synthetase | Chl biosynthesis | [6] |
| 2 | C4PW04 | MPBQ | MPBQ methyltransferase | Chl biosynthesis | [5] |
| 2 | C0KTA6 | AIDP | Fructose-bisphosphate aldolase | Glycolysis | [7] |
| 2 | Q84U08 | IMI1 | Acetohydroxyacid synthase | Aminoacid biosynthesis | [8] |
| 3 | C7ENF7 | HSP70+ | 70 kDa heat shock protein | Chaperone | [9] |
| 3 | Q41578 | PORA | protochlorophyllide reductase | Chl biosynthesis | [10] |
| 4 | Q9M4V3 | RCAB+ | Ribulose bisphosphate carboxylase activase B | Calvin cycle | [11] |
| 4 | Q6XW17 | PSB 10kD | Photosystem II, subunit 10kD | Electron transport | [12] |
| 4 | P69555 | PSBH | Photosystem II, subunit H | Electron transport | [13] |
| 4 | P11383 | RBCL+ | Ribulose bisphosphate carboxylase large chain | Calvin cycle | [14] |
| 5 | Q5J331 | APX+ | Thylakoid ascorbate peroxidase | Photoprotection, H_2_O_2_ | [15] |
| 5 | Q5XUV3 | PSB29 | Photosystem II, Inositol phosphatase-like protein | Biogenesis of photosystem II | [16] |
| 5 | P56761 | PSBD+ | Photosystem II, subunit D | Electron transport | [13] |
| 6 | I0JTV1 | UNKNOWN |  |  |  |
| 6 | P62720 | YCF4 | hypothetical chloroplast open reading frame 4 | Assembly of photosystem I | [17] |
| 6 | P69386 | PSBE | Photosystem II, subunit E | Electron transport | [18] |
| 7 | P24064 | CLPP+ | ATP-dependent Clp protease proteolytic subunit | Proteolysis | [19] |
| 7 | Q00434 | PSBP | Photosystem II, subunit P | Electron transport | [20] |
| 8 | P80602 | BAS1 | 2-Cys peroxiredoxin | Photoprotection, H_2_O_2_ | [21] |
| 8 | AT5G49910 | HSC70-7 | 70 kDa heat shock protein | Chaperone | [9] |
| 8 | P26302 | KPPR | Phosphoribolukinase | Calvin cycle | [22] |
| 9 | P12112 | ATPA+ | ATP synthase, subunit A | ATP synthesis | [23] |
| 9 | P20858 | ATPB+ | ATP synthase, subunit B | ATP synthesis | [23] |
| 9 | D3K4D8 | ATPG | ATP synthase, subunit G | ATP synthesis | [23] |
| 9 | P17933 | RPS2 | Ribosome, small ribosomal subunit 9 | Translation | [24] |
| 9 | P06528 | ATPF | ATP synthase, subunit F | ATP synthesis | [23] |
| 9 | F1DKC1 | CAT | Catalase | Photoprotection H_2_O_2_ | [25] |
| 10 | Q9FRZ5 | RBCS | Ribulose bisphosphate carboxylase small chain | Calvin Cycle | [14] |
| 10 | F8WTQ5 | TAALAD1a | Delta-aminolevulinic acid dehydratase | Chl biosynthesis | [26] |
|  | AT1G06430 | FTSH8 | Zn metalloprotease 8 | Photosystem II repair | [27] |
|  | A4UQP4 | PSBQlike | Photosystem II, subunit Q like | Electron transport | [28] |
| 12 | P05151 | PETA+ | Cytochrome b6f, subunit f | Electron transport | [29] |
| 12 | Q7X9A6 | PETC | Cytochrome b6f, iron sulfur subunit | Electron transport | [30] |
| 12 | P69443 | ATPE | ATP synthase, subunit E | Electron transport | [23] |
| 12 | ATCG00720 | PETB+ | Cytochrome b6f, subunit b6 | Electron transport | [29] |
|  | Q95H46 | NDHF | NAD(P)H-quinone oxidoreductase, subunit F | Electron transport | [31] |
|  | Q8L803 | RPL9 | Ribosome, large subunit 9 | Translation | [24] |
| 13 | Q95H42 | NDHH | NAD(P)H-quinone oxidoreductase, subunit H | Electron transport | [31] |
| 13 | Q95H60 | NDHJ | NAD(P)H-quinone oxidoreductase, subunit J | Electron transport | [31] |
| 13 | Q8RVZ8 | FNR+ | Ferredoxin-NADP(H) oxidoreductase | Electron transport | [32] |
|  | Q5S1S6 | PRXQ | Peroxiredoxin Q | Photoprotection, H_2_0_2_ | [33] |
|  | Q45FE7 | GGR+ | Geranylgeranyl hydrogenase | Chl Biosynthesis | [34] |

1. Sattler SE, Cahoon EB, Coughlan SJ, DellaPenna D. Characterization of tocopherol cyclases from higher plants and cyanobacteria. Evolutionary implications for tocopherol synthesis and function. Plant Physiology. 2003;132(4):2184-95. Epub 2003/08/13. PubMed PMID: 12913173; PubMed Central PMCID: PMC181302.

2. Dong H, Deng Y, Mu J, Lu Q, Wang Y, Xu Y, et al. The Arabidopsis Spontaneous Cell Death1 gene, encoding a zeta-carotene desaturase essential for carotenoid biosynthesis, is involved in chloroplast development, photoprotection and retrograde signalling. Cell Res. 2007;17(5):458-70. Epub 2007/05/01. doi: 10.1038/cr.2007.37. PubMed PMID: 17468780.

3. Blecher O, Erel N, Callebaut I, Aviezer K, Breiman A. A novel plant peptidyl-prolyl-cis-trans-isomerase (PPIase): cDNA cloning, structural analysis, enzymatic activity and expression. Plant Mol Biol. 1996;32(3):493-504.

4. Haga K, Iino M. Phytochrome-mediated transcriptional up-regulation of ALLENE OXIDE SYNTHASE in rice seedlings. Plant Cell Physiol. 2004;45(2):119-28. Epub 2004/02/28. PubMed PMID: 14988482.

5. Bollivar DW. Recent advances in chlorophyll biosynthesis. Photosynth Res. 2006;90(2):173-94. Epub 2007/03/21. PubMed PMID: 17370354.

6. Hirel B, Vidal J, Gadal P. Evidence for a cytosolic-dependent light induction of chloroplastic glutamine synthetase during greening of etiolated rice leaves. Planta. 1982;155(1):17-23. Epub 1982/06/01. doi: 10.1007/bf00402926. PubMed PMID: 24271621.

7. Rutter WJ. EVOLUTION OF ALDOLASE. Fed Proc. 1964;23:1248-57. Epub 1964/11/01. PubMed PMID: 14236133.

8. Pang SS, Duggleby RG, Guddat LW. Crystal structure of yeast acetohydroxyacid synthase: a target for herbicidal inhibitors. Journal of Molecular Biology. 2002;317(2):249-62. Epub 2002/03/21. doi: 10.1006/jmbi.2001.5419. PubMed PMID: 11902841.

9. Kiang JG, Tsokos GC. Heat shock protein 70 kDa: molecular biology, biochemistry, and physiology. Pharmacol Ther. 1998;80(2):183-201. Epub 1998/12/05. PubMed PMID: 9839771.

10. Heyes DJ, Hunter CN. Making light work of enzyme catalysis: protochlorophyllide oxidoreductase. Trends Biochem Sci. 2005;30(11):642-9. Epub 2005/09/27. doi: 10.1016/j.tibs.2005.09.001. PubMed PMID: 16182531.

11. Portis AR, Jr. Rubisco activase. Biochim Biophys Acta. 1990;1015(1):15-28. Epub 1990/01/04. PubMed PMID: 2404515.

12. Stockhaus J, Hofer M, Renger G, Westhoff P, Wydrzynski T, Willmitzer L. Anti-sense RNA efficiently inhibits formation of the 10 kd polypeptide of photosystem II in transgenic potato plants: analysis of the role of the 10 kd protein. EMBO J. 1990;9(9):3013-21. Epub 1990/09/01. PubMed PMID: 1697267; PubMed Central PMCID: PMC552019.

13. Komenda J, Reisinger V, Muller BC, Dobakova M, Granvogl B, Eichacker LA. Accumulation of the D2 protein is a key regulatory step for assembly of the photosystem II reaction center complex in Synechocystis PCC 6803. Journal of Biological Chemistry. 2004;279(47):48620-9. doi: 10.1074/jbc.M405725200. PubMed PMID: WOS:000225098100018.

14. Garcia-Murria MJ, Karkehabadi S, Marin-Navarro J, Satagopan S, Andersson I, Spreitzer RJ, et al. Structural and functional consequences of the replacement of proximal residues Cys(172) and Cys(192) in the large subunit of ribulose-1,5-bisphosphate carboxylase/oxygenase from Chlamydomonas reinhardtii. Biochem J. 2008;411(2):241-7. Epub 2007/12/13. doi: 10.1042/bj20071422. PubMed PMID: 18072944.

15. Caverzan A, Bonifacio A, Carvalho FE, Andrade CM, Passaia G, Schunemann M, et al. The knockdown of chloroplastic ascorbate peroxidases reveals its regulatory role in the photosynthesis and protection under photo-oxidative stress in rice. Plant Sci. 2014;214:74-87. Epub 2013/11/26. doi: 10.1016/j.plantsci.2013.10.001. PubMed PMID: 24268165.

16. Keren N, Ohkawa H, Welsh EA, Liberton M, Pakrasi HB. Psb29, a conserved 22-kD protein, functions in the biogenesis of Photosystem II complexes in Synechocystis and Arabidopsis. Plant Cell. 2005;17(10):2768-81. Epub 2005/09/13. doi: 10.1105/tpc.105.035048. PubMed PMID: 16155179; PubMed Central PMCID: PMC1242271.

17. Schottler MA, Albus CA, Bock R. Photosystem I: its biogenesis and function in higher plants. J Plant Physiol. 2011;168(12):1452-61. Epub 2011/01/25. doi: 10.1016/j.jplph.2010.12.009. PubMed PMID: 21255865.

18. Pakrasi HB, Williams JG, Arntzen CJ. Targeted mutagenesis of the psbE and psbF genes blocks photosynthetic electron transport: evidence for a functional role of cytochrome b559 in photosystem II. EMBO J. 1988;7(2):325-32. Epub 1988/02/01. PubMed PMID: 3130246; PubMed Central PMCID: PMC454321.

19. Sjogren LL, Stanne TM, Zheng B, Sutinen S, Clarke AK. Structural and functional insights into the chloroplast ATP-dependent Clp protease in Arabidopsis. Plant Cell. 2006;18(10):2635-49. Epub 2006/09/19. doi: 10.1105/tpc.106.044594. PubMed PMID: 16980539; PubMed Central PMCID: PMC1626633.

20. Nishimura T, Uno C, Ido K, Nagao R, Noguchi T, Sato F, et al. Identification of the basic amino acid residues on the PsbP protein involved in the electrostatic interaction with photosystem II. Biochim Biophys Acta. 2014;1837(9):1447-53.

21. Dietz KJ. Peroxiredoxins in plants and cyanobacteria. Antioxid Redox Signal. 2011;15(4):1129-59. Epub 2011/01/05. doi: 10.1089/ars.2010.3657. PubMed PMID: 21194355; PubMed Central PMCID: PMC3135184.

22. Hariharan T, Johnson PJ, Cattolico RA. Purification and characterization of phosphoribulokinase from the marine chromophytic alga Heterosigma carterae. Plant Physiology. 1998;117(1):321-9. Epub 1998/05/22. PubMed PMID: 9576802; PubMed Central PMCID: PMC35018.

23. Kinosita K, Jr., Yasuda R, Noji H, Adachi K. A rotary molecular motor that can work at near 100% efficiency. Philos Trans R Soc Lond B Biol Sci. 2000;355(1396):473-89. Epub 2000/06/03. doi: 10.1098/rstb.2000.0589. PubMed PMID: 10836501; PubMed Central PMCID: PMC1692765.

24. Brodersen DE, Nissen P. The social life of ribosomal proteins. The FEBS journal. 2005;272(9):2098-108. Epub 2005/04/28. doi: 10.1111/j.1742-4658.2005.04651.x. PubMed PMID: 15853795.

25. Willekens H, Chamnongpol S, Davey M, Schraudner M, Langebartels C, Van Montagu M, et al. Catalase is a sink for H2O2 and is indispensable for stress defence in C3 plants. EMBO J. 1997;16(16):4806-16. Epub 1997/08/15. doi: 10.1093/emboj/16.16.4806. PubMed PMID: 9305623; PubMed Central PMCID: PMC1170116.

26. Beale SI, Gough SP, Granick S. Biosynthesis of delta-aminolevulinic acid from the intact carbon skeleton of glutamic acid in greening barley. Proc Natl Acad Sci U S A. 1975;72(7):2719-23. Epub 1975/07/01. PubMed PMID: 1058487; PubMed Central PMCID: PMC432842.

27. Zaltsman A, Ori N, Adam Z. Two types of FtsH protease subunits are required for chloroplast biogenesis and Photosystem II repair in Arabidopsis. Plant Cell. 2005;17(10):2782-90. Epub 2005/08/30. doi: 10.1105/tpc.105.035071. PubMed PMID: 16126834; PubMed Central PMCID: PMC1242272.

28. Calderone V, Trabucco M, Vujicic A, Battistutta R, Giacometti GM, Andreucci F, et al. Crystal structure of the PsbQ protein of photosystem II from higher plants. EMBO Rep. 2003;4(9):900-5. Epub 2003/09/02. doi: 10.1038/sj.embor.embor923. PubMed PMID: 12949587; PubMed Central PMCID: PMC1326360.

29. Gray JC. Cytochrome f: Structure, function and biosynthesis. Photosynth Res. 1992;34(3):359-74. Epub 1992/12/01. doi: 10.1007/bf00029811. PubMed PMID: 24408832.

30. Jahns P, Graf M, Munekage Y, Shikanai T. Single point mutation in the Rieske iron-sulfur subunit of cytochrome b6/f leads to an altered pH dependence of plastoquinol oxidation in Arabidopsis. FEBS Lett. 2002;519(1-3):99-102. Epub 2002/05/23. PubMed PMID: 12023025.

31. Trost P, Bonora P, Scagliarini S, Pupillo P. Purification and properties of NAD(P)H: (quinone-acceptor) oxidoreductase of sugarbeet cells. Eur J Biochem. 1995;234(2):452-8. Epub 1995/12/01. PubMed PMID: 8536688.

32. Hu P, Lv J, Fu P, Hualing M. Enzymatic characterization of an active NDH complex from Thermosynechococcus elongatus. FEBS Lett. 2013;587(15):2340-5. Epub 2013/06/01. doi: 10.1016/j.febslet.2013.05.040. PubMed PMID: 23722112.

33. Lamkemeyer P, Laxa M, Collin V, Li W, Finkemeier I, Schottler MA, et al. Peroxiredoxin Q of Arabidopsis thaliana is attached to the thylakoids and functions in context of photosynthesis. The Plant journal : for cell and molecular biology. 2006;45(6):968-81. Epub 2006/03/02. doi: 10.1111/j.1365-313X.2006.02665.x. PubMed PMID: 16507087.

34. Tanaka R, Rothbart M, Oka S, Takabayashi A, Takahashi K, Shibata M, et al. LIL3, a light-harvesting-like protein, plays an essential role in chlorophyll and tocopherol biosynthesis. Proceedings of the National Academy of Sciences of the United States of America. 2010;107(38):16721-5. doi: 10.1073/pnas.1004699107. PubMed PMID: ISI:000282003700061.
