# Supplementary material for: Lil3 Assembles with Proteins Regulating Chlorophyll Synthesis in Barley
Source: PLoS One. 2015 Jul 14;10(7):e0133145. doi: 10.1371/journal.pone.0133145 (PMC4501709; doi:10.1371/journal.pone.0133145)
Supplement: S2 Table — (DOCX) [file pone.0133145.s006.docx]

**Table S2. Interaction analysis of POR, CHS, Lil3, and GGR.**

| **Prey** | **Bait** | | | |
| --- | --- | --- | --- | --- |
|  | **CHS** | **Lil3.1** | **Lil3.2** | **GGR** |
| **PORA** | **-** | **+** | **+** | **+** |
| **PORB** | **-** | **+** | **+** | **+** |
| **PORC** | **-** | **+** | **+** | **+** |
| **CHS** | **+** | **+** | **+** | **+** |
| **Lil3.1** | **+** | **/** | **/** | **-** |
| **Lil3.2** | **+** | **/** | **/** | **-** |
| **GGR** | **-** | **-** | **-** | **-** |

NMY51cells were co-transformed with BTC-*Lil3:1* or BTC-*Lil3:2* and, PRN-*PORA*, PRN-*PORB*, PRN-*PORC* PRN-*CHS,* PRN*-*GGR, and with BTC-*GGR or* BTC-*CHS* and, PRN-*PORA*, PRN-*PORB*, PRN-*PORC*, PRN*-GGR*, PRN-*CHS,* PRN-*Lil3:1,* PRN-*Lil3:2*. Expression of protein pairs (prey in column, bait in row) that result in growth of yeast under suppressing growth conditions are labelled with a plus (+). The absence of growth is labelled with a minus (-). Protein pairs not tested are labelled with a slash (/).
